# A New Cryptic Species of the Genus *Mychonastes* (Chlorophyceae, Sphaeropleales)

**DOI:** 10.3390/plants11233363

**Published:** 2022-12-03

**Authors:** Nikita Martynenko, Evgeniy Gusev, Dmitry Kapustin, Maxim Kulikovskiy

**Affiliations:** 1 A.N. Severtsov Institute of Ecology and Evolution, Russian Academy of Sciences, Leninsky Prospect 33, 119071 Moscow, Russia; 2 K.A. Timiryazev Institute of Plant Physiology, Russian Academy of Sciences, Botanical Street 35, 127276 Moscow, Russia

**Keywords:** *Mychonastes*, new species, SSU rDNA, ITS2 rDNA secondary structure, CBC approach, Moscow

## Abstract

A new species of green coccoid algae, *Mychonastes hindakii* sp. nov., was isolated from the River Moscow (Russia, Moscow). The taxon is described using morphological and molecular methods. *Mychonastes hindakii* sp. nov. belongs to the group of species of the genus *Mychonastes* with spherical single cells joined with mucilaginous, irregularly shaped stalks. A comparison of ITS2 rDNA sequences and its secondary structures combined with the compensatory base changes approach confirms the separation between *Mychonastes hindakii* and other species of the genus. *Mychonastes hindakii* sp. nov. represents a cryptic species that can only be reliably identified using molecular data.

## 1. Introduction

The term ‘(pseudo)cryptic taxa’ is commonly referred to as a complex of more or less morphologically indistinguishable taxa that are clearly distinguishable genetically. The cryptic species are known from different algal lineages, e.g., diatoms [1,2], chrysophytes [3,4], euglenoids [5,6], chlorophytes [7,8] and streptophytes [9,10].

The algal genus *Mychonastes* comprises unicellular or colonial algae with spherical, ovoid, or ellipsoidal cells with or without a mucilage envelope. Each cell contains 1–4 discoid or mostly cup-shaped chloroplasts without a pyrenoid. Colonies consist of 4–64 or more cells. Cells are in colonies that are connected by gelatinous strands formed by cell wall remnants of the mother cell [11]. Originally, it was described as a monotypic genus with *M. ruminatus* Simpson and Van Valkenburg as its type [12]. Later, three species were added [13,14]. Then, Krienitz et al. [11] revised the genus based on morphological observations and SSU and ITS rRNA sequence analyses, described eight new species and transferred the species from the genus *Pseudodictyosphaerium* Hindák (=*Dictyosphaeriopsis* Hindák) to *Mychonastes*. Recently, Patova et al. [15] described another new species from a cold-water stream in the subpolar Urals.

The members of the genus *Mychonastes* are widely distributed throughout the world inhabiting lotic and lentic waters, soils, and aerial habitats [11,16]. For instance, the type species, *M. ruminatus*, was isolated from the brackish water of Chesapeake Bay (Maryland, USA) [12]. Although *M. homosphaera* was described from freshwaters, it is considered one of the most common green algal species in soils [16,17]. Actually, strain *M. homosphaera* QUCCCM70, isolated from desert soil in Qatar, showed a high antioxidant capacity [18]. Additionally, *Mychonastes* can dominate in freshwater picoplankton of lakes [19]. Selected strains of some *Mychonastes* species (e.g., *M. afer*, *M. homosphaera* and *M.* spp.) are a potential feedstock for biodiesel and feed production [18,20,21,22].

The aim of this article is to report our observations on the *Mychonastes* strain isolated from the Moscow River (Moscow, Russia) and describe it as a new species.

## 2. Results

**Figure 1:**
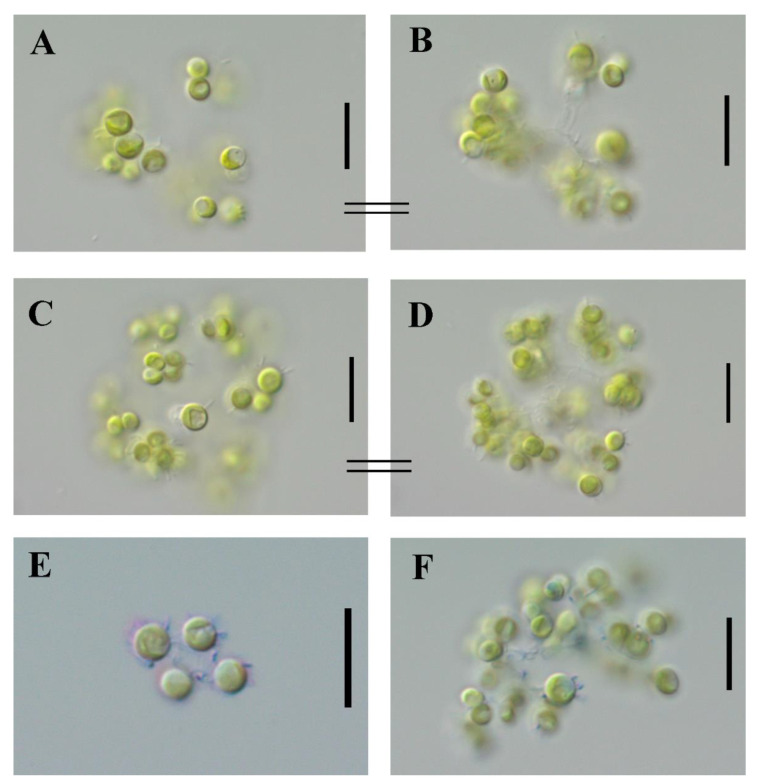
*Mychonastes hindakii* sp. nov. (**A**–**D**). Unstained colonies. (**E**,**F**) Colonies stained with methylene blue. Scale bars: 10 μm.

Cells of the strain M323 are small, spherical, and commonly in colonies connected by strands with mucilaginous envelopes. Stalks between cells are visible with light microscopy. The cellular and colony morphologies of the isolate suggested that it is related to the genus *Mychonastes* (Figure 1).

**Figure 2:**
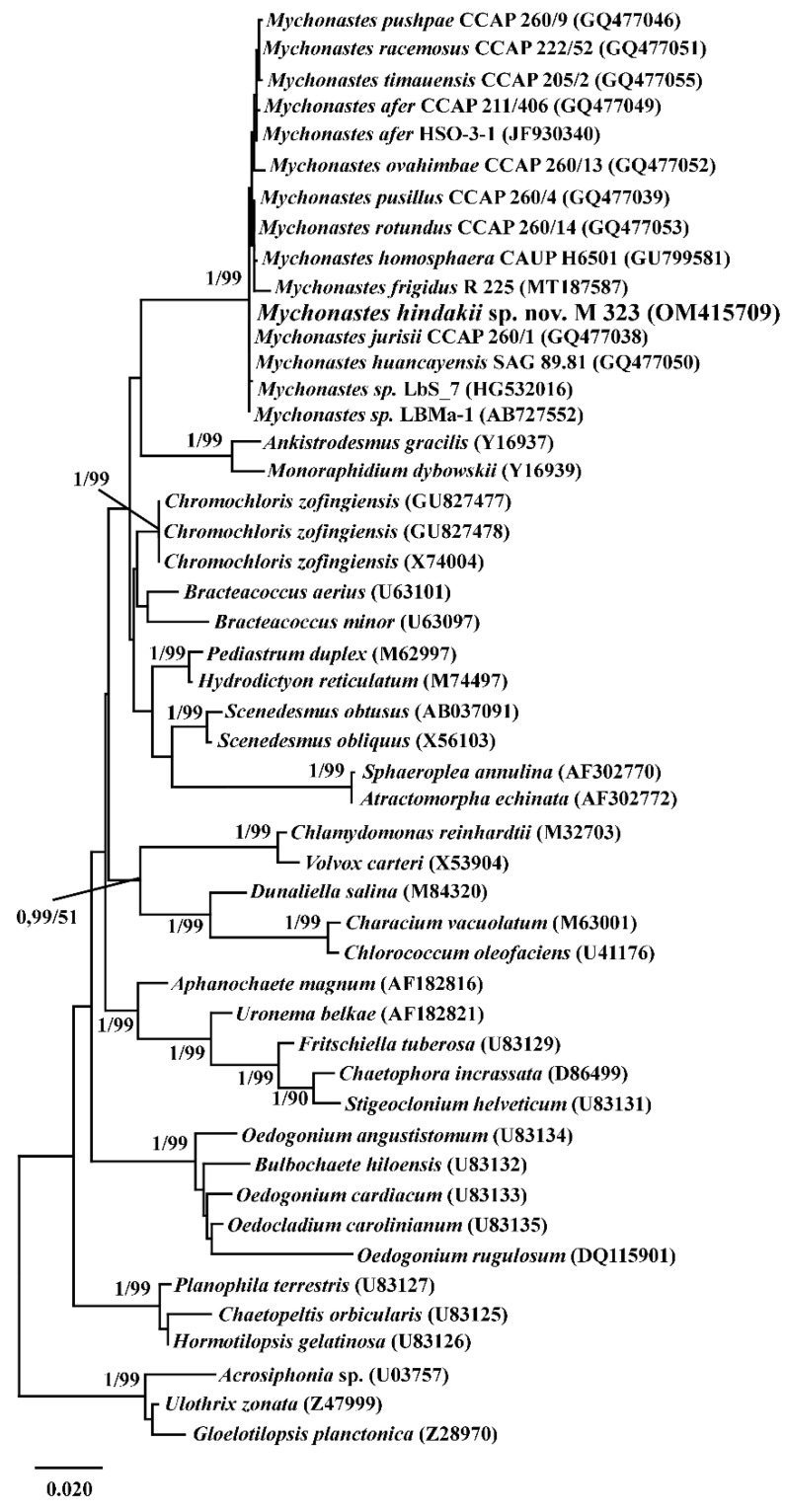
Maximum likelihood tree of the partial small subunit rDNA (SSU rDNA). The Bayesian posterior probability and maximum likelihood bootstrap values are shown to the left and right of the fraction line respectively. Scale bar represents the substitution per site.

In the analysis of the SSU rDNA gene sequences, strain M323 belongs to the *Mychonastes* clade on the phylogenetic tree of Chlorophyceae with maximum values of Bayesian posterior probability and maximum likelihood (ML) bootstrap (Figure 2).

**Figure 3:**
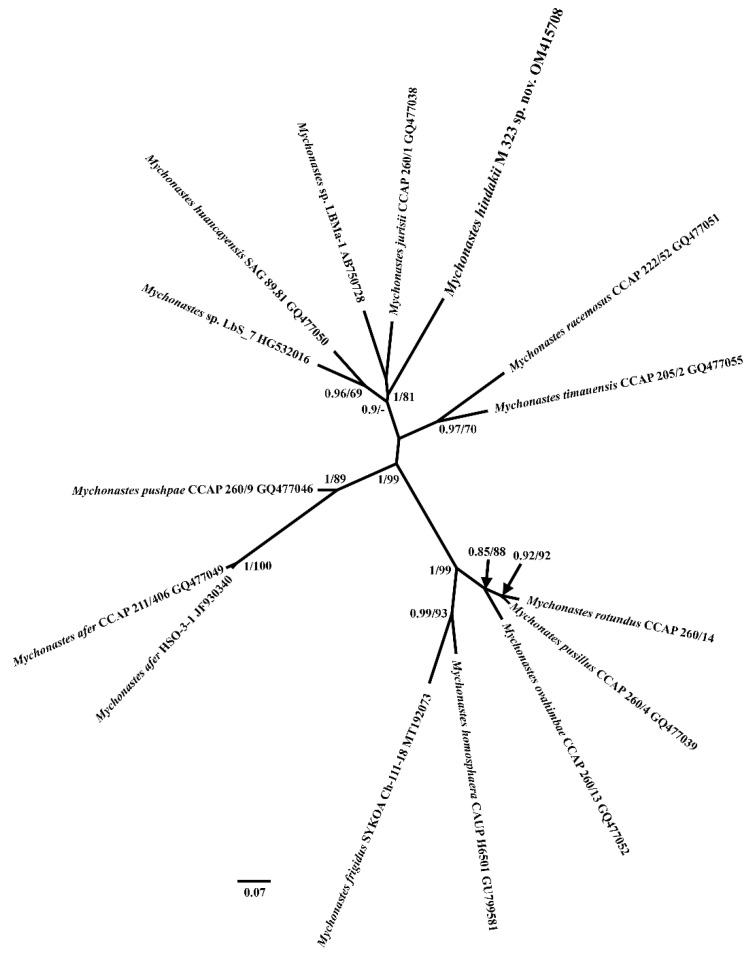
Unrooted maximum likelihood tree of the ITS2 rDNA of species from the genus *Mychonastes*. The Bayesian posterior probability and maximum likelihood bootstrap values are shown to the left and right of the fraction line respectively. Scale bar represents the substitution per site.

Morphological and molecular analyses confirmed that strain M323 belongs to the *Mychonastes* genus. The strain M323 belongs to the morphological group with species *M. racemosus* Krienitz et al., *M. jurisii* (Hindák) Krienitz et al., *M. pushpae* Krienitz et al., *M. afer* Krienitz et al. and *M. ovahimbae* Krienitz et al. [3], which have cells in colonies, joined by stalks. Species in this group can be unambiguously distinguished by the presence of CBCs (Compensatory Base Changes) in ITS2 sequences [3]. Analysis of the ITS2 rDNA of strain M323 allowed the recognition of the new lineage on the ITS2 rDNA tree (Figure 3).

This strain forms its own branch with high values of Bayesian posterior probability and ML bootstrap (1/81). The ITS2 sequence data show that *M. hindakii* is included in a clade that consists of two morphologically different species: *M. jurisii* and *M. huancayensis* Krienitz et al. [11]. This cluster also included two unidentified symbiotic strains LbS_7 [23] and LBMa-1 [24].

Here, we describe this lineage as a new species.

Phylum **Chlorophyta** Reichenbach.

Class **Chlorophyceae** Wille.

Order **Sphaeropleales** Luerssen.

Genus ***Mychonastes*** Simpson and Van Valkenburg.

***Mychonastes hindakii*** Martynenko, Gusev, Kapustin and Kulikovskiy sp. nov. (Figure 1).

**Diagnosis:** Colonies have 4-8-16-32 cells, which are 17–37 μm in diameter with mucilaginous envelopes. Cells are spherical, 3–4.4–(5.7) μm in diameter and connected via mucilaginous, irregularly shaped stalks. The chloroplast is single, parietal, and cup-shaped, without a pyrenoid. Propagation can be by two or four autospores. The species differs from other species of the genus by the order of the nucleotides in ITS rDNA gene sequences.

*Holotype (here designated):* a large drop of unfixed dried cells of the strain M323 on watercolor paper, deposited at the Herbarium of the Main Botanical Garden, Russian Academy of Sciences, Moscow, Russia (MHA) under the designation MHA M323.

*Authentic strain:* M323.

*Etymology:* The specific epithet is dedicated in honour of the prominent Slovak phycologist Prof. DrSc. František Hindák (1937–2019) for his contribution to the taxonomy of coccoid green algae.

*Type locality:* The Moscow River, Moscow, Russia. Latitude/Longitude: 55°47′28″ N, 37°24′53″ E, elevation 123 m above sea level. Collected and isolated on 28 July 2019 by N. Martynenko.

*Representative DNA sequences:* GenBank accession numbers OM415708 (nuclear 5.8S-ITS2-LSU rDNA), and OM415709 (nuclear SSU-ITS1-5.8S rDNA).

*Distribution:* To date, *Mychonastes hindakii* has been observed in this type locality. At the time of collection, the pH was 7.03, the temperature was 19.9 °C, and the specific conductance was 347 μS/cm. To date, *Mychonastes hindakii*, except the type locality, was also found widespread in Europe. Molecular signatures of ITS1 of this species were spotted in 31 lakes of Europe in Austria, France, Germany, Hungary, Italy, Poland, Romania, Spain, and Sweden [25]. Some characteristics of the lakes where *M. hindakii* was found by molecular signatures are presented in Table 1.

## 3. Discussion

Currently, the genus *Mychonastes* includes 21 species. The data on nucleotide sequences are only known for 11: *M. afer*, *M. frigidus* Patova et al., *M. homosphaera* (Skuja) Kalina and Punčochářová, *M. huancayensis*, *M. jurisii*, *M. ovahimbae*, *M. pusillus* Krienitz et al., *M. pushpae*, *M. racemosus*, *M. rotundus* Krienitz et al., and *M. timauensis* Krienitz et al. The remaining 10 species, including generitype (*M. anomalus* (Korshikov) Krienitz et al., *M. botrytella* (Komárek and Perman) Krienitz et al., *M. densus* (Hindák) Krienitz et al., *M. deiccatus* S.W. Brown, *M. elegans* (Bachmann) Krienitz et al., *M. fluviatilis* (Hindák) Krienitz et al., *M. lacunaris* (Hindák) Krienitz et al., *M. minusculus* (Hindák) Krienitz et al., *M. ruminatus* and *M. scoticus* (Hindák) Krienitz et al.) have not been sequenced yet. Molecular data demonstrated that the *Mychonastes* clade represents a monophyletic lineage within Chlorophyceae, which is clearly differentiated from other evolutionary lineages containing small-celled coccoid green algae [11].

Like many other green algae, morphological convergence and high levels of morphological plasticity complicate the application of traditional classification methods in this genus. For morphological delineation of species in the genus, features such as form and size of cells, colony-forming or solitary cells, presence or absence of mucilaginous stalks and their branching patterns (regular or irregular), presence or absence of mucilaginous envelope are usually used [11]. However, most of these features are variable in culture. Under culture conditions, most colonial forms can disintegrate, thus presenting challenges for their differentiation not only from *Mychonastes* species with solitary cells, but from other single small-celled, spherical or ellipsoidal chlorophytes such as *Choricystis* (Skuja) Fott, *Meyerella* Fawley and K. Fawley, or *Nannochloris* Naumann [26,27,28,29,30]. The ITS2 sequence data did not support discrimination between the colonial and single-celled *Mychonastes* species. It is considered that the shape of mucilaginous stalks might have some taxonomic value but it seems that their shape can significantly vary in culture. For instance, Hindák [31] illustrated a strain of *Mychonastes jurisii* that has colonies with both regular mucilaginous stalks (such as in *M. timauensis*) and irregular ones. Based on these data, Krienitz et al. [11] stated that only molecular data can provide conclusive evidence for the characterization and separation of species of the genus *Mychonastes*.

The ITS2 region has an important role in rRNA processing because it contains specific cleavage sites for further elimination. The process of excising ITS2 in the RNA transcript requires the formation of the correct ITS2 secondary structures, which are conserved across most eukaryotes [32,33]. ITS2 secondary structures are often used for delimiting biological species based on the presence of compensatory base changes (CBCs) [34,35]. The CBC species concept states that two organisms/strains whose ITS2 sequences differ by even a single CBC in the conserved regions of Helix II and Helix III represent two different biological species [34]. According to Müller et al. [36] and Wolf et al. [37], the presence of any CBC in the whole ITS2 molecule is sufficient for distinguishing species. The CBC concept was successfully used for species delimitation in different and independent groups of algae such as cryptophytes [38,39], diatoms [40], eustigmatophytes [41] and green algae [42]. Comparing the secondary structure of ITS-2, compensatory base changes are found to discriminate new species in the *Mychonastes* clade too [11,15].

**Figure 4:**
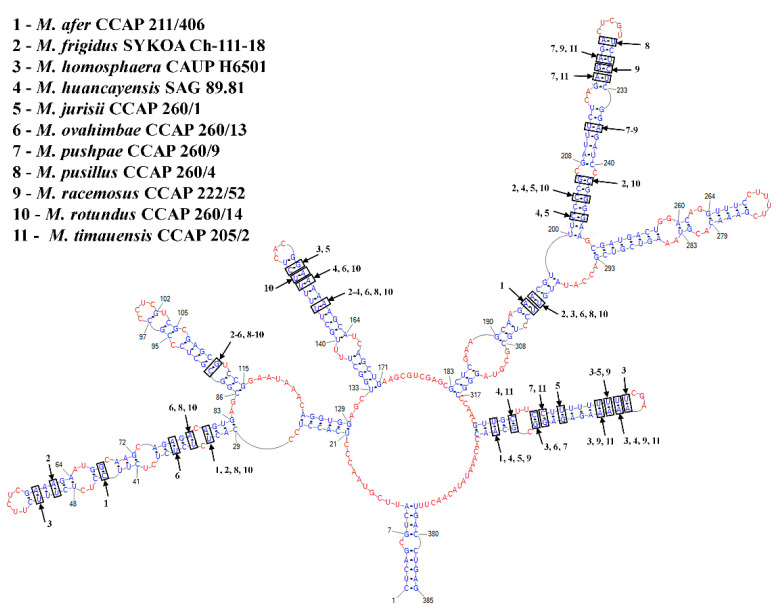
Predicted secondary structure of the nuclear internal transcribed spacer 2 of the strain M323 (*Mychonastes hindakii* sp. nov.). Black boxes indicate the positions of Compensatory Base Changes (CBCs) between *M. hindakii* and other species of the genus.

All *Mychonastes* taxa are characterized by variability in the length and shape of the ITS2 secondary structure. *M. hindakii* has a 367 bp ITS2 molecule length. It is the longest in comparison with relative species *M. jurisii* (319 bp) and *M. huancayensis* (328 bp), but shorter than M. *frigidus* and *M. pusillus* (383 bp). To take into account the difference in length and a high degree of variability, we used the secondary structure alignment for CBCs search and unrooted ITS2 tree recovering. Comparison of the ITS2 secondary structures of *M. hindakii* with other species with known ITS2 rDNA sequences revealed a number of CBCs with all studied taxa, including highly conserved areas of Helix III (Figure 4).

Therefore, *M. hindakii* differs from other species with a considerable number of CBCs—up to 10. The new species has as a minimum of one Compensatory Base Change in comparison with each *Mychonastes* species in the third helix and a minimum of four in the full structure, but the more significant CBC is situated in the fourth pair position in helix Ib because this helix is almost conservative in other known species of *Mychonastes* and lacks structure changes. *M. hindakii* differs in this position from each *Mychonastes* species, except *M. afer*, *M. pushpae* and *M. timauensis*, which have a hemi-CBC in this position. Despite high levels of CBCs, the number of hemi-CBCs was not so high and was equal to *M. hindakii* and several different species. It differed from one in *M. huancayensis, M. jurisii, M. pushpae* and *M. racemosus* to five in *M. homosphaera*, *M. pusillus* and *M. rotundus.* The estimated genetic divergence (p-distances) was the highest (0.190) between *M. hindakii* and *M. frigidus* and lowest (0.128) between *M. pushpae* (Table 2).

*Mychonastes hindakii* clustered with colonial *M. jurisii* and unicellular *M. huancayensis* on the unrooted ITS2 tree and formed an individual branch (Figure 3). The clade, consisting of two morphologically different species, was also recovered in previous ITS2 analyses [11,15], which contradicts the importance of cell organization in the systematics of *Mychonastes.* Morphologically, *Mychonastes hindakii* is most similar to *M. jurisii*. This species was originally described as *Dactylosphaerium jurisii* Hindák [31], however, since the type species of *Dactylosphaerium*, *D. sociale* Steinecke is multiplied by zoospores and has chloroplasts with a pyrenoid, *D. jurisii* was transferred to *Pseudodictyosphaerium* [43]. Later, Krienitz et al. [11] transferred all *Pseudodictyosphaerium* species to *Mychonastes*. *Mychonastes jurisii* has a slightly greater cell diameter range, 2–6 μm, whereas *M. hindakii* cells have a diameter of 3–4.4 μm. Additionally, according to the original description, colonies of *Mychonastes jurisii* have 4-8-16 cells, whereas colonies in *M. hindakii* consist of 4-8-16-32 cells. The most obvious difference in these species is seven differences in the ITS2 structure (CBCs), one in Ib and II helices, two in III helix, and three in IV helix.

Unlike *Mychonastes hindakii*, *M. huancayensis* forms generally solitary cells. Additionally, the cell diameter is significantly larger in *M. huancayensis* than in *M. hindakii* (4–10 μm vs. 3–4.4 μm, respectively). Between *M. hindakii* and *M. huancayensis* there are nine CBCs: one in Ib helix, per two in II and III helices, and four in IV helix. There is one additional important difference between these species; unlike the famous and widespread *M. jurisii*, the second sister species, *M. huancayensis*, is known only from Peru [11].

The cluster of the described species also included two unidentified strains LbS_7 and LBMa-1, which are symbiotic for a different group of protists. The strain LbS_7 was isolated from primmorphs of the endemic Baikal Lake freshwater sponge *Lubomirskia baicalensis* Pallas (Lubomirskiidae, Porifera). This strain has a simple morphology with solitary cells, such as *M. homosphaera,* which is not enough for the assignment of this strain to any described species [23]. Molecular data, obtained from the strain, show crucial differences from other strains, and probably, it represents a new species, which has not been described yet. The second strain, LBMa-1, is a photobiont of *Stentor polymorphus* (Müller) Ehrenberg (Heterotrichea, Ciliophora). Cells of this strain in culture were solitary, without a mucilaginous covering, lacking inter-connecting threads between cells, and spherical or ovoid (2.8–3.4 vs. 4.1–4.8 μm) [24]. This strain does not have significant differences in the nucleotide composition of ITS2 rDNA and its secondary structure from *M. jurisii*, thus, it refers to this species.

Other colonial species with nucleotide sequence data, *Mychonastes afer*, *M. ovahimbae* and *M. pushpae*, have the same cell diameter range (2–5 μm), which overlaps with the cell diameter range of *M. hindakii* (3–4.4 μm), but they differ considerably from *M. hindakii* by the ITS2 sequences, secondary structure, and CBC number (Table 2, Figure 3 and Figure 4). *Mychonastes afer* has four CBCs in comparison with new species, *M. ovahimbae*, and *M. pushpae* have seven and five CBCs, respectively. Moreover, these taxa have some minute morphological differences. Both *Mychonastes afer* and *M. ovahimbae* have four-celled colonies and *M. pushpae* has 4-8-16 celled colonies, whereas *M. hindakii* colonies consist of 4-8-16-32 cells. Additionally, *M. hindakii* can be differentiated from all these species by the mucilaginous stalk shape. In both *Mychonastes afer* and *M. ovahimbae,* the mucilaginous stalks are tattered, in *M. pushpae,* the mucilaginous stalks are irregularly flattened, and in *M. hindakii,* they are irregularly shaped.

Moreover, *Mychonastes hindakii* is similar to *M. racemosus* but the latter species has a greater cell diameter range (2.5–6 μm) and larger colonies (10–80 μm). The main morphological distinction of *Mychonastes hindakii* from *M. timauensis* is that, in the latter species, the cells in colonies are connected by mucilaginous, regularly shaped, broad and flat rhomboid stalks, while in *M. hindakii,* the stalks are irregularly shaped. Additionally, *M. timauensis* colonies are larger (8–60 μm). As with the previous taxa, *M. racemosus* and *M. timauensis* also differ from new species by CBCs; nine with the first and six with the latter species, respectively.

Among species without nucleotide sequence data, *Mychonastes hindakii* can be compared with *M. minusculus* and *M. lacunaris*. *Mychonastes minusculus*, however, has smaller cells (1.8–3.7 μm in diameter) than *M. hindakii*. In contrast to *M. lacunaris*, *M. hindakii* has irregularly shaped mucilaginous stalks. Additionally, *M. hindakii* is somewhat similar to the specimens reported by Hindák from a fishpond in Western Slovakia and identified as *Dictyosphaerium botrytella* Komárek and Perman *forma* [44]. The mature cells of Slovak specimens were spherical to spherical–oval, 4–6 × 3.5–5 μm. Unfortunately, the true taxonomic identity of those Slovak specimens cannot be resolved. It is very likely that they represent a separate, yet undescribed species but they definitely do not belong to *D. botrytella* in terms of cell shape and size. Now, *D. botrytella* is a member of *Mychonastes* [11].

Our analysis confirms the widespread distribution of *M. hindakii* across European water bodies. Molecular signatures of the ITS1 of this species were found in 31 lakes of Europe in Austria, France, Germany, Hungary, Italy, Poland, Romania, Spain, and Sweden [25]. The new species tolerates a wide range of environmental parameters. *Mychonastes hindakii*, like other species of the genus, can be unambiguously identified only with molecular data.

## 4. Materials and Methods

### 4.1. Study Area

The sample was collected from the Moscow River (55°47′28″ N, 37°24′53″ E, 123 m above sea level) on 28 July 2019. The studied area is located in the central part of the East European Plain, in Moscow city, at the territory of the natural monument, Park Serebryany Bor. The climate of Moscow city is moderately continental with an average February temperature of −6.7 °C and an average +19.2 °C in July. Annual precipitation is 600–800 mm [45]. The Moscow River has a longitude of 473 km, and the water basin is nearly 17,600 km^2^. The river accounts 61% of the snow supply, up to 27% of the underground water supply, and 12% of the rain supply [46].

### 4.2. Samples and Collection

Planktonic samples were collected by N. Martynenko using a plankton net with 20 μm mesh size. Water mineralization and temperature measurements were performed using the Hanna device (HI 9828, Hanna Instruments, Inc., Woonsocket, RI, USA). The water temperature at the time of collection was 19.9 °C, pH = 7.03, and the specific conductance was 347 μS/cm. Strain was isolated, and culture was perpetually transferred to the Collection of Algae at Laboratory of Molecular Systematics of Aquatic Plants of K.A. Timiryazev Institute of Plant Physiology of the Russian Academy of Sciences (IPP RAS), also was deposited in the Culture Collection of Microalgae at IPP RAS under the number M 323 and Culture Collection of Algae of the Institute of Biology of Komi Science Centre under the number SYKOA Ch-166-21.

### 4.3. Culturing

The sample was placed immediately in the field in a sterile Waris-H medium [47]. Monoclonal strain was established by examination of micro-pipetted single cells under an inverted microscope. Non-axenic unialgal cultures were maintained in Waris-H liquid medium at 10 °C in a growth chamber with a 12:12 h light:dark photoperiod with 50–100 mmol photons m^–2^ s^–1^. Morphological features were investigated after cultivation on 3N BBM, WC, BG-11 liquid and agar mediums [16,48].

### 4.4. Light Microscopy

Light microscopy observations and photography were carried out using a Zeiss Scope A1 microscope (Carl Zeiss AG, Oberkochem, Germany) and Nikon Eclipse80i (Nikon, Tokyo, Japan) with optics of Nomarski (DIC), equipped with an oil immersion objective (Plan-Apochromat 100x/1.40 Oil DIC M27) with magnification up to 1000×. Cells were stained with 0.1% methylene blue solution and 1.0% ink solution for the determination of the mucilage structure. Cultures were observed in exponent and stationary growth phases. Light micrographs were taken with an AxioCam ERc 5s Rev.2 and Nikon Digital Sight Ds—2Mv cameras.

### 4.5. Extraction of DNA and Amplification

The total DNA of the monoclonal culture was extracted using InstaGeneTM Matrix according to the manufacturer’s protocol. Fragments of SSU-ITS1 rRNA (2076 bp) were amplified using pairs of primers: 18S-F [49] and 18L [50] for SSU rRNA fragments, and SR10 [51] and ITS2_broad R [25] for the 3′-end of SSU, including the V9 region, and ITS1 rRNA fragments. Fragment of partial 5.8S-ITS2-partial LSU rDNA (753 bp) was amplified using a pair of primers crITS_03F, crITS_05R [52].

Amplification of all studied fragments was carried out using the premade mix ScreenMix (Evrogen, Russia) for the polymerase chain reaction (PCR). The conditions of amplification for SSU rRNA, V9 SSU-ITS1 rRNA, and 5.8S-IST2-LSU rDNA fragments were: an initial denaturation of 5 min at 95 °C, followed by 35 cycles at 94 °C for denaturation (30 s), 52 °C for annealing (30 s) and 72 °C for extension (50–80 s), and a final extension of 10 min at 72 °C. The resulting amplicons were visualized by horizontal agarose gel electrophoresis (1.5%), colored with SYBR Safe (Life Technologies, Carlsbad, CA, USA). Purification of DNA fragments was performed with the ExoSAP-IT kit (Affymetrix, Santa Clara, CA, USA) according to the manufacturer’s protocol. All studied fragments were decoded from two sides using forward and reverse PCR primers and the Big Dye system (Applied Biosystems, Foster City, CA, USA), followed by electrophoresis using a Genetic Analyzer 3500 sequencer (Applied Biosystems, Foster City, CA, USA). For phylogenetic analysis fragments were used separately, containing only partial SSU rDNA, and only ITS2 rDNA.

### 4.6. Phylogenetic Analysis of SSU rDNA Data

Sequences were edited manually and assembled using BioEdit v7.1.3, and MegaX [53]. Newly determined sequences and GenBank sequences of 43 other chlorophytes from different morphological groups were included in the alignment. Additionally, two species, *Ulothrix zonata* (F. Weber et Mohr) Kützing and *Gloeotilopsis planctonica* M.O.P. Iyengar and Philipose, were chosen as outgroup taxa. Sequences were aligned using global SILVA alignment for rRNA genes in the SINA v1.2.11 [54]. jModelTest ver. 2.1.10 [55], using the Bayesian information criterion (BIC), indicated that the TrN model of nucleotide substitution, with Gamma (G) distributed rates across sites and a proportion of invariable sites (I), was the most appropriate evolutionary model for the SSU rDNA alignment. Maximum likelihood phylogeny (ML) was constructed using MegaX [53] with the Subtree–Pruning–Regrafting tree rearrangements algorithm (SPR). The bootstrap analysis with 1000 replicas was used. Bayesian Inference (BI) analysis was conducted with MrBayes-3.2.5 [56], using SYM+G+I model. Three “hot” and one “cold” Markov chains were run for 15 × 10^6^ cycles in two repetitions with the selection of each 100th generated tree. Phylogenetic tree and posterior branching probabilities were obtained after discarding the first 25% to produce estimate parameter models of nucleotide substitutions and likelihood. Viewing and editing of trees were carried out in the programs FigTree (ver 1.4.2) and Adobe Photoshop CC (19.0).

### 4.7. Internal Transcribed Spacer 2 Annotation, Secondary Structure Modeling, Alignment and Phylogeny

For the annotation of the ITS2 sequence, we used ITS2-Annotation [57]. This tool uses HMMer [58] to annotate ITS2 sequences with Hidden Markov Models (HMMs) [59]. It returns, by definition of the ITS2, the sequence between the conserved 5.8S and 28S (or equivalent) rRNA funding region. The secondary structure of the ITS2 rDNA region was modeled using the Predict a Secondary Structure Web Server (http://rna.urmc.rochester.edu; accessed on 21 November 2020). This server combines four separate prediction and analysis algorithms: calculating a partition function, predicting a minimum free energy (MFE) structure, finding structures with maximum expected accuracy, and pseudoknot prediction. The construction of the model accounted for the presence of the pyrimidine–pyrimidine unpaired section in the second helix at the seventh position [60], as well as the length and nucleotide composition of the spacers in the core of the model defining the helix boundaries [33]. Detail features of the ITS2 secondary structures of the studied species, such as the presence of helices Ib and IIIb, were also checked using secondary structures of other *Mychonastes* species [11]. The resulting secondary structure was visualized in PseudoViewer3 [61]. The ITS2 sequences were aligned according to the secondary structure by ClustalW algorithm [62] in 4SALE [63]. Delimitations of species secondary structures of *Mychonastes* strains were checked for compensatory base changes (CBCs) according to Coleman [34,35] and Müller et al. [36] in comparison with species described by Krienitz et al. [11] and Patova et al. [15]. For the phylogenetic analysis, the sequence part from the sequence–structure alignment (as obtained by 4SALE) was further processed using the same programs and methods as described above for the 18S data, except for the model of nucleotide substitution—HKY+G+I.

### 4.8. Blast Search of Barcoding Data (ITS1)

To assess the distribution of described species, we carried out a blastn search of its internal transcribed spacer 1 sequence in the database, containing data of metabarcoding studies of 218 European freshwater lakes presented by Boenigk et al. [25] In this study, we used a 300 nt region of ITS1-5.8S rDNA as a representative for delineation of *Mychonastes* species. The search was carried out in all 436 SRA experiments with program megablast and 5000 target sequences. We considered only matches with 99% similarity, i.e., sequences with no more than two changes (substitution, indel) in comparison with the initial sequence.

## Figures and Tables

**Table 1:** Number and characteristics of lakes where *Mychonastes hindakii* was reported based on metabarcoding data of Boenigk et al. [25] and our data *.

Country	No. lakes	pH, $\frac{\max}{\min}$	Conductivity, $\frac{\max}{\min}$	Temperature °C, $\frac{\max}{\min}$	Altitude, $\frac{\max}{\min}$
Austria	2	$\frac{8.3}{8.2}$	$\frac{691}{469}$	$\frac{25.5}{21.1}$	$\frac{417}{221}$
France	2	$\frac{9.2}{8.2}$	$\frac{227}{80}$	$\frac{26.2}{24.1}$	$\frac{323}{190}$
Germany	13	$\frac{10}{7.7}$	$\frac{689}{112}$	$\frac{24.0}{19.5}$	$\frac{450}{-3}$
Hungary	1	9.1	587	28.0	144
Italy	1	8.6	220	27.2	64
Poland	5	$\frac{9.8}{7.9}$	$\frac{538}{279}$	$\frac{26.5}{20.3}$	$\frac{169}{76}$
Romania	1	8.5	334	24.5	542
* Russia	1	7.0	347	19.9	123
Spain	2	$\frac{8.6}{7.5}$	$\frac{674}{241}$	$\frac{23.7}{20.8}$	$\frac{207}{88}$
Sweden	4	$\frac{8.9}{7.5}$	$\frac{361}{52}$	$\frac{19.6}{18.2}$	$\frac{61}{43}$

**Table 2:** Number of CBCs/ hemi-CBCs in ITS2 and based on this marker p-distances between *M. hindakii* M 323 (367 nt) and other species of the genus.

№	Taxa	Helix Ia	Helix Ib	Helix II	Helix IIIa	Helix IV	∑CBC	p-Distance	ITS2 Length
1	*M. afer* CCAP 211/406	2/1	0/1	0/0	1/1	1/0	4/3	0.176	322
2	*M. frigidus* SYKOA Ch-111-18	2/1	1/1	1/0	3/1	0/0	7/3	0.190	383
3	*M. homosphaera* CAUP H6501	1/1	1/1	2/2	1/1	5/0	10/5	0.182	338
4	*M. huancayensis* SAG 89.81	0/0	1/0	2/0	2/0	4/1	9/1	0.138	328
5	*M. jurisii* CCAP 260/1	0/0	1/1	1/0	2/0	3/0	7/1	0.129	319
6	*M. ovahimbae* CCAP 260/13	2/0	1/0	2/0	1/2	1/1	7/3	0.155	362
7	*M. pushpae* CCAP 260/9	0/0	0/1	0/0	3/0	2/0	5/1	0.128	354
8	*M. pusillus* CCAP 260/4	2/0	1/0	1/2	3/2	0/1	7/5	0.173	383
9	*M. racemosus* CCAP 222/52	0/0	1/0	0/0	3/1	4/0	8/1	0.168	380
10	*M. rotundus* CCAP 260/14	2/0	1/0	3/1	3/2	0/2	9/5	0.151	380
11	*M. timauensis* CCAP 205/2	0/0	0/1	0/1	2/0	4/2	6/4	0.131	351

## Data Availability

Not applicable.
